# A NEW STEP TO PREVENT ELDER ABUSE IN JAPAN

**DOI:** 10.1093/geroni/igac059.1358

**Published:** 2022-12-20

**Authors:** Noriko Tsukada, Naoki Ikeda, Asako Katsumata

**Affiliations:** Nihon University, Tokyo, Tokyo, Japan; Kamihonnmachi sougou houritsu jimusyo, Tennouji-ku, Osaka, Japan; Rehabilitation Institution, Chiba, Chiba, Japan

## Abstract

This paper first introduces major aspects of the Japanese Act on the Prevention of Elder Abuse, including trends in substantiated elder abuse cases in both institutional and domestic settings, definitions of elder abuse, and abusers and reporting systems. It then introduces the recent amendment of operational standards in institutional settings made by ministerial ordinance. The amendment includes a requirement to develop a committee to prevent occurrence and recurrence of elder abuse and to appoint a designated individual to establish guidelines and implement training. It is hoped to further prevent elder abuse in Japan.

